# Vitalism and Vital Medication

**Published:** 1877-06

**Authors:** R. C. Bond

**Affiliations:** Class ’57, Aurora, Ind.


### ART. 2.—VITALISM AND VITAL MEDICATION.

*A Paper read before the Alumni Association of the Miami Medical College, February 26th, 1877,*

BY R. C. BOND, M. D., CLASS '57, AURORA, IND.

In considering this subject, I have purposely omitted psychological phenomena and special therapeutics, not because of their irrelevancy, but on account of a lack of sufficient time to do justice to those interesting features of my subject.

That the physician has to deal with the most abstruse and important problems in the whole range of human affairs, we think, will bear the test of honest and enlightened criticism. Life in its broadest sense, is a phenomena of transcendent interest, and is clothed with mysteries surpassing all other subjects of human thought.

Life, or Vitalism, in its primordeal essence is unknown and probably unknowable by any means within the range of man's perceptions, and possibly for lack of knowledge of the nature and essential demands of this principal or force, we are ever to remain in total ignorance, not only as to how it attaches to matter, but of any *infallible* means of prolonging and sustaning its vital operations in the organism beyond a determinate allotment.

As to vitality, we concede that it can be investigated only as correlative with matter; that inheres or prevades the germinal element, or bioplasm of living structures; that it is autonomous, or self-moving and self-directing in the presence of a suitable pabulum; that it may be acted upon by external agents through the media of its own production; and that it does, by the brunt of adverse physical forces or conditions, cease to hold its correlative place in the organism.

We conceive vitality, in its essentiality, to be totally distinct from the physical forces, though intimately connected there-with; that it is *autonomic* and determines such a molecular disposition as is essential to the elaboration of the organism, and that it also conserves those molecular conditions and motions essential to every vital process. We dissent from the position that the physical forces, as light, heat, electricity, etc., are correlated as the organizing force of vital acts; also that there exists no *super*-terrestrial force, called vital, as a determining and controlling force in the organization of the living body and its complex activities.

We can not conceive that inorganic matter and the physical forces, can be in any sense, other than only adjuvants of life, and are intended by the Supreme Architect to subserve the demands and ends of life on the globe. We think it more reasonable to conclude with the learned R. P. Secchi, that "life is a phenomena of an entirely different order from the rest of the cosmogony—it is germinal, and pre-supposes certain powers which can not be obtained by a simple combination affected according to the laws which control the molecules of inorganic matter."

We also maintain that observation and reason bear us out in the assumption that the objective end of the cosmogony was life, and that life is in no sense, an accidental or fortuitous phenomenon of the physical forces and inorganic matter. These, though prior in the order of time, or subordinate in importance and design, to life the chief end of the cosmos. Inorganic matter and the physical forces are simply *elements*

of the machinery, so to speak, of life. The most careful analytic and synthetic experimentations, as well as every process of philosophical and syllogistic induction, have utterly failed to give any definite or satisfactory solution of the phenomenon of life, based upon the hypothesis of a correlation of the physical forces.

To ignore, or overlook the fact of life, or vitalism, as an entity differing from all other principles or forces in nature, is to lose sight of the plainest lesson of experience and reason.

Finding no analogue of vitalism in any simple principle in nature, or in any other combination affected according to the laws which control inorganic matter, it is reasonable to conclude that life is a *super*-physical force of an entirely different order from the rest of the cosmogony. To accept this, is the only way to escape the *stifling maze* into which some modern biologists have fallen; for, no lessons of experience, or of inductive or deductive reason, afford us any other legitimate clue to the origin and distinctive principle of life.

Whether this vitalizing principle or force becomes extinct upon the death of the organism, or is resolved to a germinal state or condition, to be re-correlated with matter in the far-off future, are questions that may be asked, but not answered by physical or scientific tests.

*Pathological view.* That pathological, or diseased conditions occur in the essential nature of vitality, as some pathological phenomena, denominated vital, would seem to indicate, may be regarded as problematical, more especially by those biologists who maintain that life is a sequence of organization. But as an individual opinion, we hold that such conditions do not obtain, but that the whole history of life, in the organism, is one of persistent integrity in its essentiality, and that its varying phenomena are from sources entirely relative.

We freely admit degrees of its manifestations, as seen in individual dynamia, adynamia, and other peculiarities of ancestral or fortuitous origin, or as may be observed in disease, or exhaustive efforts of body and mind, or as in inanition. Nor, in this view of vitalism, will it be necessary to deny that it may be in embryo, full development, or decline, as these relative states of latency or activity do not effect its essential nature.

But we do assume that its apparent abnormities, as well as its uncertain tenure, are due to a perverted or changed pabulum, unsuited to its normal work, and the laws of an indefinite existence.

In premature death, vital extinction is evidently due to structural or organic changes, and is it at all unreasonable to presume that the decline, or decrepitude of old age is the result wholly of mal-adjustment between nutrition and disintegration, and not from an inherent waning of iife in its essentiality ?

By mal-adjustment, we mean, that the normal processes of nutrition and retrograde metamorphosis in advancing age, become by slow degrees, less and less synchronous, resulting in pectous changes in the colloidal structures cf the organism, hence the shrinking and diminished elasticity of old age.

Why these processes were *so gaged*, or the reason of this lack of synchronism, is hidden with the Divine Law-Giver, whose wisdom and out-look are too infinite to call in question The primal impulse, whether dust or otherwise, that determines organization, or if you please, concourse of atoms, is the principle or force we denominate vital, and which we claim to be, not *nisus consecutions*, but the *nisus formations* of organization. Such a force, in the nature of things must exist, otherwise we have effects without a cause. A cause must of necessity pre-exist, and be an entity, in some important sense, independent of its effects. To presume then that the vital force or principal may be morbid, *per se*, seems much like assuming that there may be pathological oxygen or hydrogen, or of any other ultimate principles.

Permit me to suggest, by the way, that diseases of an adynamic type, and all maladies indicating asthenia, or approaching death, are such by reason of the vital force being held in *abeyance* by structural or functional conditions of the organism ; and that death, somatic and molecular, is but the *smothering out*, so to speak, of the vital force, and not, we conceive, from disease or death of vitality itself, as first in the order of causation. In this view we get a clue to the reason why doubts may occur as to the exact date of absolute death, for in some modes of death the vital extinction may be the last link in the chain of that, to us, sad phenomenon.

*Vital Medication.* If the preceding views of vitality be correct, there can be no such thing as direct vital medication, in other words, life itself, so far as we can understand, needs no medication. In perverted vital force, no remedies can be more than proximate or relative.

Life or vitalism, in disease affecting its normal play, can be restored only by means, the effects of which are vital liberation and conservation. Such a view may be thought, by some, uniqe or heterodox, but by the way of compensation or redress, allow me to assume :

1st. That the neural system is the direct medium and agent of all centric and peripheral vital acts.

2nd. That many local, as well as general maladies are due to perverted nerve influence.

These propositions are not at all new, but by reason of their practical bearings should be clearly registered upon the mind.

These assumptions, for my present purpose, will be sufficiently substantiated by referring to the fact of the intimate connection and dependence of life upon the integrity of the cerebro-spinal centres ; being the axis of all sensory and motor phenomena, whether automatic or volitional, thus making it at once apparent, how much attaches to nervous integrity in every vital process.

In a transmitted impression or force, the state or condition of the conducting medium becomes a matter essential to such result. This is strictly true in physics, nor, can it be less so, according to physiological law.

That morbific agents do produce such molecular changes or conditions in nervous structures as to modify or change their normal impressibility and force, we think will not be denied, for disease of the nervous tissue is as well established as any fact in pathological anatomy. And from the office and functions of the neural system, it is not unreasonable to conclude that many non-cognizable morbific agents make their first and chief attack upon integrity, and thus, directly induce perverted nerve force, while other *materies morbi* indirectly produce similar results.

Future research in biology may *possibly* give us more definite and practical views as to the effects of external impressions upon the living material, or bioplasm, which by virtue of its own endowments determines every vital act ; yet, so far as we now understand, we get no centric expression except through the medium of the nervous system. We may concede that the nerves are conductors only, without possessing any inhering sensor or motor qualities ; that the living or bioplasmic element is the source, *per se*, of every vital phenomenon, yet the fact still remains that nervous integrity is essential to all normal or living processes. Hence diseased nervous structures, inducing perverted nerve influence, are competent to play an important part in trophic, as well as centric disorders. This is quite the reverse of the views held by Blumenbach and others, "that each organ is endowed by a *vita propria* peculiar to itself," and should be treated accordingly.

Properly, we all recognize the importance of looking well to organic and functional processes, and of accurately observing their reflex phenomena, yet we may fail to estimate the *pre-eminent* influence of nerve impressibility as a determining and fruitful source of numerous disorders, and the most direct avenue by which to successfully combat many local, as well as the so-called vital maladies. Neural integrity then, the axis of all vitality, is a *sine qua non* in all living processes, and to sustain nervous integrity is to conserve vital force.

